# The Effect of Implementing Mechanical Cardiopulmonary Resuscitation Devices on Out-of-Hospital Cardiac Arrest Patients in an Urban City of Taiwan

**DOI:** 10.3390/ijerph18073636

**Published:** 2021-03-31

**Authors:** Yi-Rong Chen, Chi-Jiang Liao, Han-Chun Huang, Cheng-Han Tsai, Yao-Sing Su, Chung-Hsien Liu, Chi-Feng Hsu, Ming-Jen Tsai

**Affiliations:** 1 Department of Emergency Medicine, Ditmanson Medical Foundation Chia-Yi Christian Hospital, Chiayi City 600, Taiwan; masterdoctorsky@yahoo.com.tw (Y.-R.C.); matthewstliao@yahoo.com.tw (C.-J.L.); lastar0329@gmail.com (H.-C.H.); cych03283@gmail.com (C.-H.L.); 2 Department of Emergency Medicine, Taichung Veteran’s General Hospital, Chia-Yi Branch, Chiayi City 600, Taiwan; chtsai6482@gmail.com; 3 Fire Bureau, Chiayi City Government, Chiayi City 600, Taiwan; fire001@ems.chiayi.gov.tw

**Keywords:** mechanical CPR device, out-of-hospital cardiac arrest, resuscitation, return of spontaneous circulation

## Abstract

High-quality cardiopulmonary resuscitation (CPR) is a key element in out-of-hospital cardiac arrest (OHCA) resuscitation. Mechanical CPR devices have been developed to provide uninterrupted and high-quality CPR. Although human studies have shown controversial results in favor of mechanical CPR devices, their application in pre-hospital settings continues to increase. There remains scant data on the pre-hospital use of mechanical CPR devices in Asia. Therefore, we conducted a retrospective cohort study between September 2018 and August 2020 in an urban city of Taiwan to analyze the effects of mechanical CPR devices on the outcomes of OHCA; the primary outcome was attainment of return of spontaneous circulation (ROSC). Of 552 patients with OHCA, 279 received mechanical CPR and 273 received manual CPR, before being transferred to the hospital. After multivariate adjustment for the influencing factors, mechanical CPR was independently associated with achievement of any ROSC (OR = 1.871; 95%CI:1.195–2.930) and sustained (≥24 h) ROSC (OR = 2.353; 95%CI:1.427–3.879). Subgroup analyses demonstrated that mechanical CPR is beneficial in shorter emergency medical service response time (≤4 min), witnessed cardiac arrest, and non-shockable cardiac rhythm. These findings support the importance of early EMS activation and high-quality CPR in OHCA resuscitation.

## 1. Introduction

Out-of-hospital cardiac arrest (OHCA) is a global health issue. Each year, cardiac arrest accounts for up to 3.7 million lives worldwide [1]. The incidence of OHCA has been reported to be 86.4 per 100,000 person-years in Europe, 98.1 in North America, 52.5 in Asia, and 51.1 in Taiwan [2,3]. Sustained return of spontaneous circulation (ROSC) from OHCA relies on the integral chain of survival, including early activation of the emergency medical services (EMS) system, provision of high-quality cardiopulmonary resuscitation (CPR), early defibrillation, advanced resuscitation, post-cardiac-arrest care, and recovery [4]. High-quality CPR plays a crucial role in survival of cardiac arrest [4].

High-quality and high-performance CPR has been promoted for preserving brain perfusion following ROSC as well as favorable neurological outcomes. However, there are many obstacles in pre-hospital settings that prevent the EMS system from meeting the requirements of high-quality CPR: sufficient compression rate, adequate depth, chest wall full recoil, and prevention of interruption. On the other hand, for maximizing the quality of CPR, mechanical CPR devices have been widely implemented in both pre-hospital and in-hospital settings in order to provide sufficient compression rate, adequate depth, and prevent the physical fatigue and interruption caused by manual CPR. However, there is a lack of evidence in current studies suggesting that in-hospital use of mechanical CPR devices is superior to manual CPR for acquiring ROSC or preferred neurological outcomes [5,6,7].

In pre-hospital settings, the effect of mechanical cardiopulmonary devices on patient outcome remains controversial. Randomized control trials conducted in Europe and the United States showed no difference in survival compared to manual CPR [8,9,10,11]. However, the majority of observational studies, including a recent large-scale German study, showed that mechanical CPR was associated with an increased rate of ROSC and may improve survival to hospital admission [12,13]. Mannequin studies also showed that mechanical CPR devices had higher chest compression quality during pre-hospital ambulance transport [14,15]. Hence, despite no definitive evidence of survival benefit, the application of mechanical CPR devices to reduce physical and cognitive load on emergency medical technicians (EMTs) continues to increase [16]. The results of previous studies investigating pre-hospital use of mechanical CPR devices on the outcome of patients with OHCA is summarized in Supplementary Table S1.

There are still scarce data concerning the use of mechanical CPR devices in pre-hospital settings in Asia. This study, conducted in an urban city of Taiwan, aimed to evaluate the impact of implementing mechanical CPR devices on ROSC, sustained ROSC for more than 24 h, and survival at discharge with favorable neurological outcomes in OHCA patients.

## 2. Materials and Methods

### 2.1. Study Design and Settings 

We conducted a retrospective cohort study between September 2018 and August 2020 (2 years) to evaluate the effect of mechanical CPR devices on the outcome of OHCAs in Chiayi City, Taiwan. All patients with OHCA who activated the EMS system were included in the OHCA registry of Chiayi City, a prospectively collected registry using the Utstein-style database. We obtained OHCA data from the database during the study period. Cardiac arrest was defined as the absence of signs of circulation, which was confirmed at the scene by EMTs. Patients with valid do-not-resuscitate (DNR) orders, obvious death at the scene without being transferred to hospital, aged younger than 18 years, and traumatic cardiac arrest (including hanging and drowning) were excluded from this study. The study was approved by the Institutional Review Board of the Ditmanson Medical Foundation Chia-Yi Christian Hospital (CYCH-IRB 2021016).

### 2.2. EMS in Chiayi City

Chiayi City is the second most densely populated city in Taiwan (4431.53 people per square kilometer), with an area of 60.02 km^2^ and 266,000 residents. Inhabitants over 65 years account for 16.2% of the population. There is one tertiary referral hospital, two secondary hospitals, and two primary hospitals in the city. The EMS system is based on the fire bureau and is composed of one centralized dispatch center and seven EMS stations. The EMS dispatch center functions 24-7 and is operated by experienced EMTs. Once the dispatch center is called for medical assistance, the EMTs on duty are dispatched from the nearest EMT station. The protocol to determine patients with OHCA includes a few questions asked by the dispatcher on the phone; once the patients with OHCA are identified, dispatcher-assisted cardiopulmonary resuscitation (DACPR) is initiated simultaneously. For patients with OHCA, the basic life support (BLS) protocol used by all EMTs includes CPR, defibrillation (if feasible) by an automated external defibrillator (AED), and the use of bag-valve-mask or laryngeal mask airway. If EMT paramedics (EMT-P) are present at the scene, they can provide advanced life support (ALS), including epinephrine injection and endotracheal intubation, as appropriate.

During the study period, all EMTs received regular training and performed CPR according to national guidelines based on the American Heart Association, European Resuscitation Council, and the International Liaison Committee on Resuscitation Guidelines [17,18]. The use of AEDs, maintenance of ventilation, and continued CPR during transportation to a hospital were mandatory for non-traumatic OHCA, unless ROSC was achieved. Quality control and assessment were conducted monthly to ensure resuscitation quality.

### 2.3. Device and Implementation Timeline

**Figure 1:**
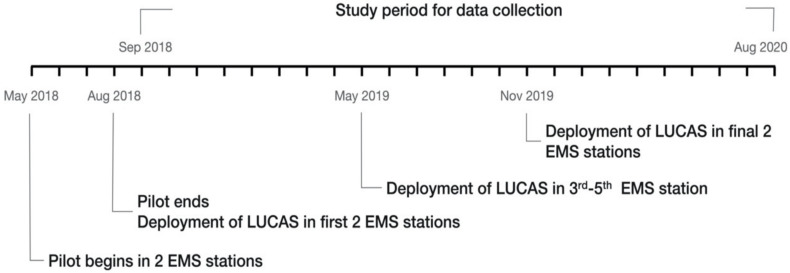
The implementation timeline of mechanical CPR device (LUCAS-2) in Chiayi EMS system.

The Lund University Cardiac Assist System-2 (LUCAS-2) chest compression system (Physio-Control Inc., Redmond, WA, USA) is a portable mechanical piston CPR device. It is battery-driven and consists of an integrated suction cup to deliver automatic chest compression and active decompression back to the neutral position of the chest. It was designed to overcome the problems identified with manual chest compressions. The LUCAS-2 device assists rescuers by delivering effective, consistent and continuous chest compressions as recommended in the current resuscitation guidelines [17,18]. It is to be used for performing external cardiac compressions on adult cardiac arrest patients. It is indicated to use the LUCAS device unless it cannot be safely or correctly placed on the patient’s chest or if the patient’s body size is too small or too large to fit the device. In May 2018, Chiayi City EMS had initiated a 3-month pilot evaluation of LUCAS-2. Two devices were first provided at two of the seven EMS stations (one device at each EMS station). After device-specific training and evaluation, LUCAS-2 was formally deployed as standard equipment in September 2018, and since November 2019, all seven EMS stations have been equipped with the LUCAS-2. The implementation timeline of LUCAS-2 in the Chiayi EMS system is shown in Figure 1. The aim of training and assessment criteria for usage of LUCAS-2 is to be able to install the device and start CPR with less than 10 s of interruption at the scene. Once LUCAS-2 is applied, except for the pause to check the pulse and analyze the cardiac rhythm through AED, it will continuously perform CPR from the scene to the hospital. In order to ensure that the quality meets the criteria, the course of resuscitation in each mission is reviewed on a monthly basis through recorded videos by an internal review board consisting of an experienced EMT-P and a medical director. The application of LUCAS-2 is dependent on the dispatcher’s identification of OHCA. In principle, however, LUCAS-2 is carried along with the dispatched EMTs unless OHCA has not been identified or the device is unavailable. When LUCAS-2 is not used, manual CPR is performed. LUCAS-2 usage is marked on the EMS record sheet.

### 2.4. Data Collection, Exposure, and Outcome

We obtained data from the prospectively collected OHCA registry of Chiayi City. The collected data included the required information according to the Utstein-style guidelines, such as patient demographics, EMS response time (defined as the time from the ambulance leaving the EMS station to the rescue scene), EMS scene time (defined as the time from the ambulance arriving at the scene to leaving the scene), EMS transport time (defined as the time from the ambulance leaving the scene to arriving at the hospital), identification time of OHCA by dispatcher, start time of DACPR or bystander CPR, number of dispatched EMTs, characteristics of cardiac arrest (witness status, initial cardiac rhythm recording by AED), location of cardiac arrest (home, public area, medical institution (local clinic and nursing home), during ambulance transport, and others), pre-hospital treatment (including use of mechanical CPR, ventilation support by bag-valve-mask or laryngeal mask, intravenous fluid or epinephrine injection, total number of electric shocks by AED), the level of transferred hospital, achievement of ROSC at any time, a sustained (≥24 h) ROSC, and survival at discharge with favorable neurologic status (GCS ≥ 13).

The exposures in this study were defined as the use of mechanical CPR (LUCAS-2) during the pre-hospital stage and ambulance transport. The primary outcome was achievement of ROSC. The secondary outcomes were sustained (≥24 h) ROSC and survival at discharge with favorable neurological status (GCS ≥ 13).

### 2.5. Statistical Analysis

Based on the need to detect an odds ratio of 1.8, achieve ROSC using mechanical CPR, and set a ROSC rate of 20% at baseline using a two-sided test size of 5% and a power of 80%, a total of 488 patients (244 in each group) would be required. Hence, we decided to include at least two years of data to meet this requirement.

Data of the included patients with OHCA were described and compared between the two groups, with and without the use of mechanical CPR. For continuous variables, Student’s t-test (presented as mean ± standard deviation) or Mann-Whitney U test (presented as medians (interquartile range)) was used, as appropriate, according to the data distribution. For categorical variables (presented as number (percentage)), the chi-square test was used. To evaluate the net effect of mechanical CPR on patient outcomes, a forward stepwise logistic regression analysis was conducted, with adjustments for variables with *p* value < 0.1 as derived from the univariate analysis and the documented or possible predictors. The adjusted factors included age [19,20], sex [19], EMS response and scene time [21,22], number of dispatched EMTs, DACPR or bystander CPR [23,24,25,26], witnessed arrest, shockable rhythm [26], location of arrest [27], pre-hospital epinephrine injection [28,29], different batches of EMS stations with LUCAS-2 implementation, and level of transferred hospital [30]. Subgroup analyses with adjustment of the above-mentioned factors were also conducted to evaluate the net effect of mechanical CPR on different types of OHCA patients, including witness status, initial cardiac rhythm (shockable or non-shockable), different EMS response times (≤ and >4 min), and different age groups (< and ≥65 years). Statistical significance was set at *p* < 0.05. Statistical analysis was performed using the JASP Team (2020) JASP (Version 0.14.1) computer software.

## 3. Results

### 3.1. Patient Population and Demographic Characteristics

**Figure 2:**
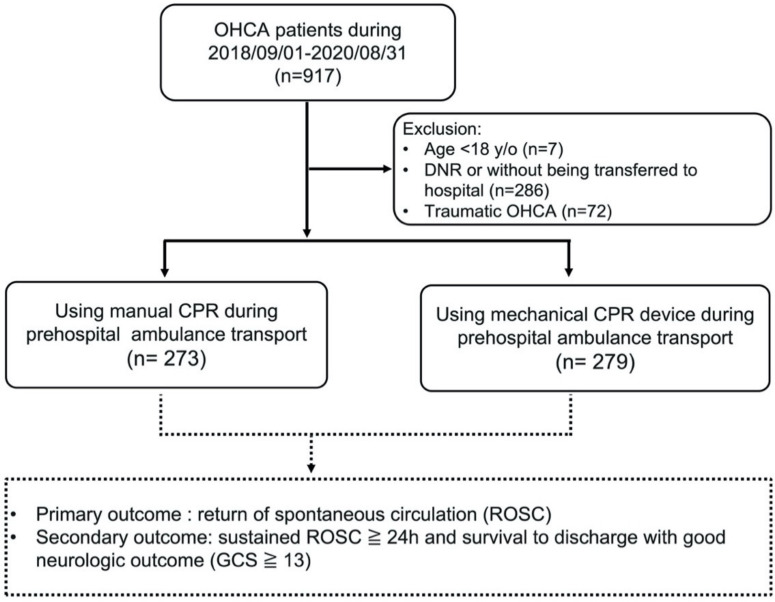
Flow chart of the patients included in the study.

Between 1 September 2018 and 31 August 2020, 917 patients with OHCA who had activated the EMS system were identified. After excluding seven patients younger than 18 years of age, 286 patients either with DNR or not transferred to the hospital, and 72 patients with traumatic causes of cardiac arrest, a total of 552 patients with OHCA were included (Figure 2). Their median and mean age were 77 (64–86) and 73.4 (±16.1) years, respectively. Among them, 72.6% were older than 65 years of age and 55.4% were male. Witnessed cardiac arrests had occurred in 50.9% of the patients and shockable rhythm in 23.7%. The cardiac arrests occurred mostly at home (77%). In total, 138 patients (25%) had ROSC, 102 patients (18.5%) had sustained (≥24 h) ROSC, and 27 patients (4.9%) survived at discharge with favorable neurological status (GCS ≥ 13).

Of the 552 patients, 279 (50.6%) received mechanical CPR and 273 (49.4%) received manual CPR at the pre-hospital stage (Figure 2). Table 1 shows the demographic characteristics of the patients treated with and without mechanical CPR. Compared to the manual CPR group, in the mechanical CPR group more patients received DACPR or bystander CPR (*p* < 0.001), the start time of DACPR was shorter (*p* = 0.03), more EMTs were dispatched (*p* < 0.001), the location of cardiac arrest was different (*p* < 0.001), the rate of administering mechanical CPR varied among the different batches of EMS stations that introduced LUCAS-2 (*p* < 0.001), and achievement of any ROSC (*p* = 0.044) and sustained (≥24 h) ROSC (*p* = 0.022) were higher. In addition, although not statistically significant, a shorter EMS response time (*p* = 0.075), longer EMS scene time (*p* = 0.061), and different proportion of transferred hospital levels (*p* = 0.079) were found in the mechanical CPR group than in the manual CPR group. There were no significant differences in age, sex, EMS transport time, identification time of OHCA by dispatcher, witnessed cardiac arrest, shockable rhythm, placement of laryngeal mask, intravenous fluid and epinephrine injection, total number of AED shocks, and the outcome of favorable neurologic status at discharge between the two groups (Table 1).

### 3.2. Impacts of Mechanical CPR on Primary Outcome

The net effect of mechanical CPR on the primary outcome—achievement of any ROSC—was evaluated using multivariate analysis. We adjusted for the variables with *p* values < 0.1, derived from the univariate analysis (Table 1) and the documented or possible predictors associated with outcomes of OHCA, including age, sex, EMS response and scene time, number of dispatched EMTs, DACPR or bystander CPR, witnessed cardiac arrest, shockable rhythm, location of arrest, pre-hospital epinephrine injection, different batches of EMS stations with LUCAS-2 implementation, and level of transferred hospital (Table 2). The results showed that the use of mechanical CPR devices had significantly higher odds of achieving ROSC (odds ratio (OR) = 1.871, 95% confidence interval (CI): 1.195–2.930, *p* = 0.006). Moreover, every year of increasing age (OR = 0.979, 95% CI: 0.966–0.992, *p* = 0.001), witnessed cardiac arrest (OR = 3.067, 95% CI: 1.966–4.786, *p* < 0.001), cardiac arrest in a public area (OR = 2.786, 95% CI: 1.319–5.886, *p* = 0.007), and ambulance transport (OR = 4.837, 95% CI: 1.459–16.039, *p* = 0.01) were independently associated with ROSC (Table 2). The final batch of EMS stations that introduced mechanical CPR showed a trend of lower odds of achievement of any ROSC than in the first batch of EMS stations (OR = 0.57, 95% CI: 0.318–1.020, *p* = 0.058), but the difference was not statistically significant.

### 3.3. Impacts of Mechanical CPR on Secondary Outcomes

The net effect of mechanical CPR on secondary outcomes, i.e., achievement of sustained (≥24 h) ROSC (Table 3) and survival at discharge with favorable neurologic status (GCS ≥ 13) (Table 4), was evaluated by multivariate analysis after adjusting for influencing factors. The use of mechanical CPR devices showed a significant association with achievement of sustained (≥24 h) ROSC (OR = 2.353, 95% CI: 1.427–3.879, *p* < 0.001) (Table 3). Every year of increasing age (OR = 0.984, 95% CI: 0.970–0.998, *p* = 0.023), every minute of increasing EMS scene time (OR = 0.939, 95% CI: 0.887–0.994, *p* = 0.029), witnessed cardiac arrest (OR = 2.069, 95% CI: 1.276–3.352, *p* = 0.003), cardiac arrest in public areas (OR = 3.187, 95% CI: 1.492–6.808, *p* = 0.003), and ambulance transport (OR = 5.527, 95% CI: 1.666–18.333, *p* = 0.005) were independently associated with sustained (≥24 h) ROSC (Table 2). However, the use of mechanical CPR devices did not show a significant association with survival at discharge with favorable neurological status (GCS ≥ 13) (OR = 1.066, 95% CI: 0.459–2.475, *p* = 0.881) (Table 4). The independent factors associated with this outcome were every year of increasing age (OR = 0.967, 95% CI: 0.945–0.989, *p* = 0.004), witnessed cardiac arrest (OR = 4.016, 95% CI:1.455–11.08, *p* = 0.007), and shockable rhythm (OR = 6.881, 95% CI: 2.844–16.653, *p* < 0.001) (Table 4).

### 3.4. Subgroup Analysis of the Effect of Mechanical CPR on Different Status of OHCA Patients

**Figure 3:**
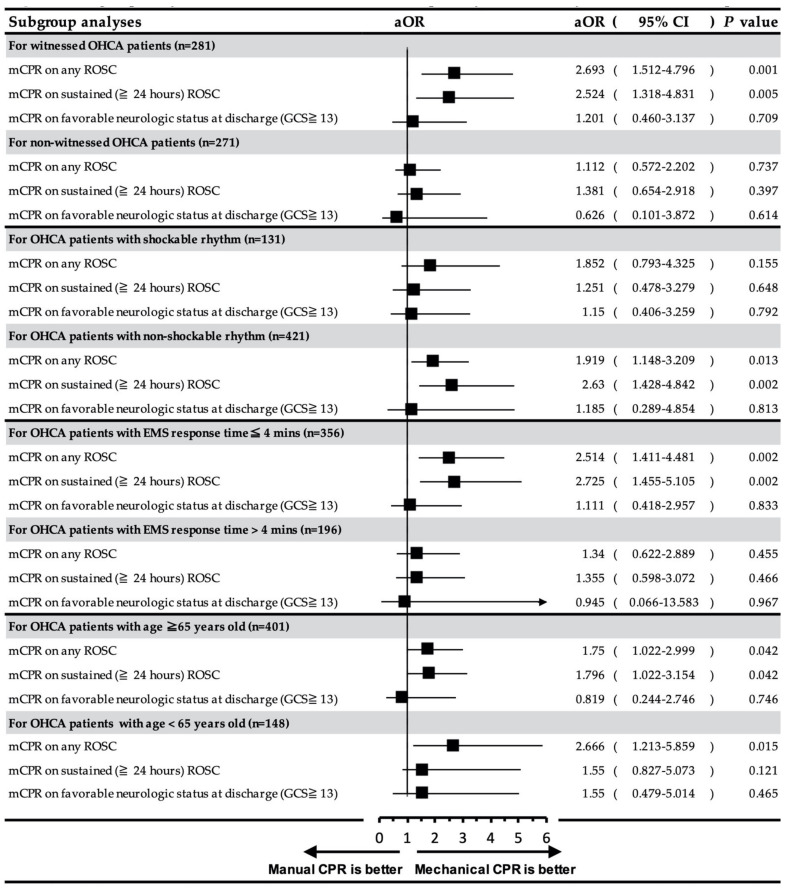
Subgroup analyses of mechanical CPR devices on the primary and secondary outcomes of OHCA patients. aOR: Adjusted odds ratio; CI: confidence interval; CPR: cardiopulmonary resuscitation; mCPR: mechanical CPR; DACPR: dispatcher-assisted CPR; BSCPR: bystander CPR; ROSC: return to spontaneous circulation; OHCA: out-of-hospital cardiac arrest.

To evaluate the net effect of mechanical CPR on the various status of OHCA patients, we performed subgroup analyses based on witness status, initial AED cardiac rhythm, different EMS response times (≤4 min and >4 min), and different age groups (<65 years and ≥65 years) (Figure 3). The possible influencing factors mentioned above were also adjusted. The results showed that mechanical CPR was independently associated with achievement of any ROSC (aOR = 2.693, 95% CI: 1.512–4.796, *p* = 0.001) and sustained (≥24 h) ROSC (aOR = 2.524, 95% CI: 1.318–4.831, *p* = 0.005) in witnessed OHCA patients but not in non-witnessed OHCA patients. Regarding the initial AED rhythm, mechanical CPR was independently associated with any ROSC (aOR = 1.919, 95% CI: 1.148–3.209, *p* = 0.013) and sustained (≥24 h) ROSC (aOR = 2.63, 95% CI: 1.428–4.842, *p* = 0.002) in OHCA patients with non-shockable rhythm but not in those with shockable rhythm. For different EMS response times, mechanical CPR showed a significant association with any ROSC (aOR = 2.514, 95% CI: 1.411–4.481, *p* = 0.002) and sustained (≥24 h) ROSC (aOR = 2.725, 95%CI: 1.455–5.105, *p* = 0.002) in OHCA patients with shorter EMS response time (≤4 min) but not in those with longer EMS response time (>4 min). Moreover, mechanical CPR was significantly associated with any ROSC (aOR = 1.75, 95% CI: 1.022–2.999, *p* = 0.042) and sustained (≥24 h) ROSC (aOR = 1.796, 95% CI: 1.022–3.154, *p* = 0.042) in patients aged ≥65 years and with any ROSC (aOR = 2.666, 95% CI: 1.213–5.859, *p* = 0.015) in patients aged <65 years.

## 4. Discussion

Our findings showed that, in OHCA patients, the use of mechanical CPR devices at the scene and during ambulance transport was associated with the achievement of any ROSC and sustained (≥24 h) ROSC, after adjustment for the independent influencing factors of ROSC (Table 2 and Table 3). During subgroup analyses, we further found that mechanical CPR devices were more effective than manual CPR in achieving ROSC, especially in patients with witnessed cardiac arrest, non-shockable rhythm, and short EMS response time (Figure 3). These findings support the importance of early EMS activation and early high-quality CPR at the pre-hospital stage.

Our study revealed that the pre-hospital use of mechanical CPR did not have any benefits regarding survival at discharge with favorable neurologic status. The independent factors most associated with this outcome were younger age, witnessed cardiac arrest, and shockable rhythm (Table 4). This finding may be reasonable because many factors can influence the outcome, such as the patient’s underlying condition, etiologies of cardiac arrest, level of the receiving hospital, in-hospital post-cardiac arrest care, and post-resuscitation care. Without appropriate control of these influencing factors, the effects of prehospital use of mechanical CPR devices may not be evident. Hence, although long-term survival with favorable neurological status would be a more relevant outcome in assessing the quality of resuscitation in OHCA patients, we set ROSC, which is a more reliable, well-recognized, and easily obtainable outcome, as the primary outcome to evaluate the effect of implementing mechanical CPR devices in our EMS system.

The resuscitative effects of mechanical CPR devices in patients with OHCA remain controversial. A previous meta-analysis pooled the studies of both the “in-hospital” and “pre-hospital” use of mechanical CPR devices and found that there was no survival benefit in using mechanical CPR devices [13,31]. However, the medical resources at in-hospital settings can be expected to be better than those in the pre-hospital setting, such as a more spacious environment, more staff for maintaining good chest compression, and more medication and equipment for advanced cardiac life support. Hence, it is reasonable that almost all studies evaluating the “in-hospital” use of mechanical CPR for OHCA patients did not show survival benefits in comparison with manual CPR [5,6,7,32]. Moreover, although large randomized controlled trials in pre-hospital settings have not shown the benefits of the routine use of mechanical CPR devices [8,9,10,11], our study results as well as the majority of the observational studies suggest an advantage over manual CPR for ROSC and survival to hospital admission [12,13,33]. When we compared the main difference between our study and previous controlled trials [9,10,11], we found an obviously shorter EMS response time (median time: 4 vs. 6–10 min), shorter total EMS time (median time: 17 vs. 36–47 min), and a higher proportion of patients receiving placement of advanced airway (82% vs. 26%) in our study (Table 1). Moreover, in our subgroup analysis, we found that the survival benefits of mechanical CPR devices were evident in patients after a shorter EMS response time (≤4 min) than after a longer EMS response time (>4 min) (Figure 3). It can therefore be assumed that the benefits of mechanical CPR emerge only when the device is applied early on-site. Further studies are required to evaluate the effect of early applied mechanical CPR devices.

In addition, our study found that the use of mechanical CPR devices was associated with ROSC, especially in witnessed cardiac arrests (Figure 3). Early high-quality CPR is important to patient outcome. In patients with non-witnessed cardiac arrests, a longer no-flow interval (duration from cardiac arrest to the start of CPR) can be expected. Therefore, even if mechanical CPR is applied to these patients, its effects on patient outcomes maybe inadequate. Furthermore, our study found that the use of mechanical CPR was more significantly associated with ROSC in patients with non-shockable rhythm than in those with shockable rhythm (Figure 3). This may be reasonable because, in OHCA patients with non-shockable rhythm, early high-quality CPR may be the most important pre-hospital factor for ROSC. In OHCA patients with shockable rhythm, in addition to high-quality CPR, early defibrillation is crucial for ROSC [4]. The relatively low number of patients with shockable rhythm (*n* = 131) in our study may have influenced the statistical power. However, although not statistically significant, we did find that the use of mechanical CPR devices in OHCA patients with shockable rhythm resulted in higher odds of achieving ROSC (Figure 3). Age is an independent factor that influences the outcomes of patients with OHCA [19,20]. In our study, every year of increasing age showed significantly lower odds of achievement of any ROSC, sustained ROSC, and survival at discharge with favorable neurologic status (Table 2, Table 3 and Table 4). A recent study in Taiwan demonstrated that pre-hospital prognostic factors in OHCA patients varied in different age groups [34]. However, in our study, we found that the use of mechanical CPR devices benefited the achievement of ROSC both in younger (<65 years) and older patients with OHCA (≥65 years) (Figure 3).

This observational study had several limitations. First, we were not able to obtain the background of the patients and their pre-existing conditions as they were sent to different hospitals. These underlying factors may influence patient outcomes. However, this occurs in most OHCA studies. Further study may be needed to control the influence of a patient’s underlying disease. Second, as with other OHCA registry analyses, we could not obtain and analyze the management that patients received at the in-hospital care stage. The post-cardiac arrest care, like targeted temperature management and coronary angiography with reperfusion therapy, are associated with a patient’s neurological outcomes. Without adjusting for these factors, the impact of pre-hospital use of mechanical CPR devices may not be evident. Instead, in order to control the factors in the in-hospital stage, we adjusted for the hospital factors based on the level of the hospital where the patients were transferred in this study. This is also the reason that we did not set the outcome of survival with favorable neurological status at discharge as our primary outcome. Third, the time of implementing LUCAS-2 in different EMS stations in our city was different; hence, the EMTs’ familiarity with the mechanical CPR device may affect the patient’s outcome. However, we adjusted for the factor of EMS stations with different times of LUCAS-2 implementation in the multivariate analysis. Finally, since this is a retrospective study, the patient’s outcome was collected from different hospital systems. There was no unified scoring system, like Glasgow-Pittsburgh cerebral performance categories, to record a patient’s neurologic status at discharge; we only could obtain the most simplified score, GCS, as the measured outcome in patient’s neurological status at discharge.

## 5. Conclusions

After a two-year period of implementing mechanical CPR devices in the EMS system, the pre-hospital use of mechanical CPR devices was significantly associated with an increased rate of ROSC in patients with OHCA. We further found that the survival benefits of mechanical CPR devices were evident in patients with a shorter (≤4 min) EMS response time and patients with witnessed cardiac arrest. These findings suggest that the benefits of mechanical CPR emerge only when the device is applied early on-site and echo that survival in cardiac arrest is highly dependent on early recognition and early application of high-quality CPR. This also may be the reason why previous controlled trials failed to show the advantages of mechanical CPR devices, because compared with this study, there was a time delay in application of the mechanical CPR devices. Hence, it is necessary to conduct further studies to evaluate the effect of early applied mechanical CPR devices on patients with witnessed cardiac arrests after appropriate adjustment of the patients’ underlying diseases and the management of post-resuscitation care.

## Figures and Tables

**Table 1:** Comparison of clinical characteristics between manual and mechanical CPR.

		Manual (*n* = 273)		Mechanical (LUCAS-2) (*n* = 279)		*p* Value
**Demographic characteristics**						
	**Age**	77.5	(63–85)	77.0	(65–86)	0.619
	**Older adults (≥65 years)**	193.0	(70.96)	208.0	(70.09)	0.275
	**Male gender**	152	(55.68)	154	(55.20)	0.910
**EMS time interval**						
	**Response time (min)**	4	(3–5)	4	(2.5–5)	0.075
	**Scene time (min)**	9	(7–12)	10	(7–12)	0.061
	**Transport time (min)**	3	(2–4)	3	(2–4)	0.194
	**Total EMS time (min)**	17	(14–20)	17	(14.5–20)	0.085
**EMS Dispatcher**						
	**DACPR or BSCPR**	131	(47.99)	197	(70.61)	<0.001
	**Identification time of OHCA by dispatcher (sec)**	63.5	(28–116.5) (*n* = 168)	58	(30–110) (*n* = 238)	0.380
	**Start time of DACPR (sec)**	199	(145–249) (*n* = 115)	167	(126–229) (*n* = 177)	0.030
	**Number of dispatched EMT (Mean ± SD)**	2.78	(0.45)	2.95	(0.33)	<0.001
	**Number of dispatched EMT (Median (IQR))**	3	(3–3)	3	(3–3)	
**Characteristics of arrest**						
	**Witnessed cardiac arrest**	146	(53.48)	135	(48.39)	0.231
	**Shockable rhythm (defibrillation)**	58	(21.25)	73	(26.17)	0.174
	**Location of arrest**					
	**Home**	198	(72.53)	227	(81.36)	<0.001
	**Public area**	24	(8.79)	17	(6.09)	
	**Medical institution**	24	(8.79)	34	(12.19)	
	**Others**	14	(5.13)	1	(0.36)	
	**During ambulance transport**	13	(4.76)	0	(0.00)	
**Pre-hospital treatment**						
	**Laryngeal mask airway**	218	(79.85)	236	(84.59)	0.146
	**Intravenous fluid injection**	10	(3.66)	17	(6.09)	0.186
	**Intravenous epinephrine**	10	(3.66)	15	(5.38)	0.333
	**Total number of AED shocks**	0	(0–0)	0	(0–1)	0.145
**Different batches of EMS stations with LUCAS-2 implementation**						
	**The first batch (2 EMS stations)**	63	(23.08)	110	(39.43)	<0.001
	**The second batch (3 EMS stations)**	124	(45.42)	114	(40.86)	
	**The final batch (2 EMS stations)**	86	(31.50)	55	(19.71)	
**Level of transferred hospital**						
	**Primary**	35	(12.82)	48	(17.27)	0.079
	**Secondary**	151	(55.31)	128	(46.04)	
	**Tertiary**	87	(31.87)	102	(36.69)	
**Outcomes**						
	**Any ROSC**	58	(21.25)	80	(28.67)	0.044
	**Sustained (≥24 h) ROSC**	40	(14.65)	62	(22.22)	0.022
	**Favorable neurologic status at discharge (GCS ≥ 13)**	12	(4.40)	15	(5.38)	0.593

Values shown are *n* (%), mean (±SD), or median (interquartile range). EMS: emergency medical services; EMT: emergency medical technician; CPR: cardiopulmonary resuscitation; DACPR: dispatcher-assisted CPR; BSCPR: bystander CPR; ROSC: return to spontaneous circulation; OHCA: out-of-hospital cardiac arrest; GCS: Glasgow Coma Scale.

**Table 2:** Predictors associated with ROSC in OHCA patients.

Parameters	OR	(95% CI)	*p* Value	aOR	(95% CI)	*p* Value
**Age (per year)**	0.978	(0.967–0.990)	<0.001	0.979	(0.966–0.992)	0.001
**Male gender**	1.103	(0.748–1.627)	0.621		-	
**EMS response time (per minute)**	0.912	(0.822–1.013)	0.085		-	
**EMS scene time (per minute)**	0.952	(0.909–0.998)	0.04		-	
**Number of dispatched EMT**	1.259	(0.770–2.060)	0.359		-	
**DACPR or BSCPR**	0.758	(0.513–1.118)	0.162		-	
**Witnessed cardiac arrest**	2.957	(1.956–4.471)	<0.001	3.067	(1.966–4.786)	<0.001
**Shockable rhythm**	1.598	(1.037–2.460)	0.033		-	
**Location of arrest**						
**Home**	reference			reference		
**Public area**	4.25	(2.205–8.191)	<0.001	2.786	(1.319–5.886)	0.007
**Medical institution**	0.957	(0.487–1.883)	0.9	0.989	(0.487–2.007)	0.976
**Others**	1.835	(0.612–5.503)	0.279	1.805	(0.556–5.866)	0.326
**During ambulance transport**	5.873	(1.876–18.383)	0.002	4.837	(1.459–16.039)	0.01
**Pre-hospital epinephrine injection**	1.735	(0.749–4.022)	0.199		-	
**Different batches of EMS stations with LUCAS-2 implementation**						
**The first batch (2 EMS stations)**	reference			reference		
**The second batch (3 EMS stations)**	0.694	(0.448–1.076)	0.102	0.794	(0.492–1.281)	0.345
**The final batch (2 EMS stations)**	0.522	(0.308–0.885)	0.016	0.57	(0.318–1.020)	0.058
**Level of transferred hospital**						
**Primary**	reference				-	
**Secondary**	0.962	(0.536–1.727)	0.898		-	
**Tertiary**	1.455	(0.799–2.647)	0.22		-	
**Mechanical CPR**	**1.49**	**(1.010–2.199)**	**0.045**	**1.871**	**(1.195–2.930)**	**0.006**

OR: odds ratio; aOR: adjusted odds ratio; CI: confidence interval; EMS: emergency medical services; EMT: emergency medical technician; CPR: cardiopulmonary resuscitation; DACPR: dispatcher-assisted CPR; BSCPR: bystander CPR; ROSC: return to spontaneous circulation; OHCA: out-of-hospital cardiac arrest.

**Table 3:** Predictors associated with sustained ROSC ≥ 24 h in OHCA patients.

Parameters	OR	(95% CI)	*p* Value	aOR	(95% CI)	*p* Value
**Age (per year)**	0.982	(0.969–0.994)	0.004	0.984	(0.970–0.998)	0.023
**Male gender**	0.884	(0.574–1.360)	0.575		-	
**EMS response time (per minute)**	0.853	(0.753–0.965)	0.012		-	
**EMS scene time (per minute)**	0.928	(0.877–0.981)	0.008	0.939	(0.887–0.994)	0.029
**Number of dispatched EMT**	1.463	(0.833–2.568)	0.185		-	
**DACPR or BSCPR**	1.128	(0.725–1.753)	0.593		-	
**Witnessed cardiac arrest**	2.347	(1.490–3.697)	<0.001	2.069	(1.276–3.352)	0.003
**Shockable rhythm**	1.82	(1.138–2.908)	0.012		-	
**Location of arrest**						
**Home**	reference			reference		
**Public area**	4.497	(2.296–8.808)	<0.001	3.187	(1.492–6.808)	0.003
**Medical institution**	1.345	(0.662–2.732)	0.413	1.33	(0.640–2.764)	0.445
**Others**	2.089	(0.645–6.768)	0.219	2.795	(0.785–9.959)	0.113
**During ambulance transport**	4.925	(1.603–15.137)	0.005	5.527	(1.666–18.333)	0.005
**Pre-hospital epinephrine injection**	1.418	(0.552–3.644)	0.469		-	
**EMS stations with different batches of LUCAS-2 implementation**						
**The first batch (2 EMS stations)**	reference				-	
**The second batch (3 EMS stations)**	0.692	(0.425–1.127)	0.139		-	
**The final batch (2 EMS stations)**	0.582	(0.325–1.042)	0.069		-	
**Level of transferred hospital**						
**Primary**	reference				-	
**Secondary**	0.848	(0.452–1.591)	0.608		-	
**Tertiary**	1.089	(0.569–2.084)	0.797		-	
**Mechanical CPR**	**1.664**	**(1.074–2.580)**	**0.023**	**2.353**	**(1.427–3.879)**	**<0.001**

OR: odds ratio; aOR: adjusted odds ratio; CI: confidence interval; EMS: emergency medical services; EMT: emergency medical technician; CPR: cardiopulmonary resuscitation; DACPR: dispatcher-assisted CPR; BSCPR: bystander CPR; ROSC: return to spontaneous circulation; OHCA: out-of-hospital cardiac arrest.

**Table 4:** Predictors associated with favorable neurologic status at discharge (GCS ≥ 13) in OHCA patients.

Parameters	OR	(95% CI)	*p* Value	aOR *	(95% CI)	*p* Value
**Age (per year)**	0.964	(0.944–0.984)	<0.001	0.967	(0.945–0.989)	0.004
**Male gender**	1.388	(0.624–3.089)	0.421		-	
**EMS response time (per minute)**	0.963	(0.787–1.180)	0.719		-	
**EMS scene time (per minute)**	0.967	(0.881–1.060)	0.471		-	
**Number of dispatched EMT**	1.536	(0.554–4.257)	0.41		-	
**DACPR or BSCPR**	1.17	(0.526–2.604)	0.701		-	
**Witnessed cardiac arrest**	4.519	(1.686–12.112)	0.003	4.016	(1.455–11.080)	0.007
**Shockable rhythm**	8.758	(3.736–20.531)	<0.001	6.881	(2.844–16.653)	<0.001
**Location of arrest**						
**Home**	reference					
**Public area**	4.655	(1.817–11.924)	0.001		-	
**Medical institution**	0.397	(0.052–3.028)	0.373		-	
**Others**		(N/A)	0.989		–	
**During ambulance transport**	1.884	(0.232–15.294)	0.553		-	
**Pre-hospital epinephrine injection**	1.746	(0.390–7.823)	0.466		-	
**EMS stations with different batches of LUCAS-2 implementation**						
**The first batch (2 EMS stations)**	reference				-	
**The second batch (3 EMS stations)**	0.968	(0.398–2.350)	0.942		-	
**The final batch (2 EMS stations)**	0.81	(0.281–2.332)	0.696		-	
**Level of transferred hospital**					-	
**Primary**	reference				-	
**Secondary**	2.14	(0.476–9.612)	0.321		-	
**Tertiary**	2.503	(0.542–11.551)	0.24		-	
**Mechanical CPR**	**1.236**	**(0.568–2.691)**	**0.594**	**1.066**	**(0.459–2.475)**	**0.881**

OR: odds ratio; aOR: adjusted odds ratio; CI: confidence interval; EMS: emergency medical services; EMT: emergency medical technician; CPR: cardiopulmonary resuscitation; DACPR: dispatcher-assisted CPR; BSCPR: bystander CPR; ROSC: return to spontaneous circulation; OHCA: out-of-hospital cardiac arrest. * Adjusting factors include age, witnessed cardiac arrest, and shockable rhythm.

## Data Availability

No new data were created or analyzed in this study.

## Supplementary Materials

The following is available online at https://www.mdpi.com/article/10.3390/ijerph18073636/s1, Supplementary Table S1: Summary of the previous studies investigating prehospital use of mechanical CPR devices on the outcome of patients with OHCA.
