# Supplementary material for: The Effect of Implementing Mechanical Cardiopulmonary Resuscitation Devices on Out-of-Hospital Cardiac Arrest Patients in an Urban City of Taiwan
Source: Int J Environ Res Public Health. 2021 Mar 31;18(7):3636. doi: 10.3390/ijerph18073636 (PMC8036320; doi:10.3390/ijerph18073636)
Supplement: Supplementary file 1 [file ijerph-18-03636-s001.pdf]

Supplementary Table 1. Summary of the previous studies investigating prehospital use of mechanical CPR devices on the outcome of patients with OHCA

Study	Study setting	Results	EMS response time (or Call to CPR) (min)	Percentage of witnessed cardiac arrest
Anantharaman 2017	RCTs	Early applied mCPR device improved ROSC and survival to hospital discharge	N/A	N/A
Axelsson 2006	Cohort study	No significant difference in ROSC and Survival to hospital discharge	10	100%
Axelsson 2013	Cohort study	No survival benifit in use of mCPR devices	9	56%
Buckler 2016	Cohort study	mCPR was associated with lower rate of ROSC and neurological favorable survival	N/A	N/A
Newberry 2018	Cohort study	No significant difference in ROSC and Survival to hospital discharge	N/A	41%
Perkins 2015	RCTs	No significant difference in ROSC and Survival to hospital discharge	6.5 (4.8-9.1)	61%
Rubertsson 2014	RCTs	No significant difference in ROSC and Survival to hospital discharge	10 (7-14)	66%
Seewald 2019	Cohort study	mCPR was associated with higher rate of ROSC	9.0 ± 6.3	56.70%
Smekal 2011	RCTs	No significant difference in ROSC and Survival to hospital discharge	7.9	69.60%
Wik 2014	RCTs	No significant difference in ROSC and Survival to hospital discharge	60% > 5 mins	47.70%
Zeiner 2015	Cohort study	No significant difference in ROSC and Survival to hospital discharge	7 (5-9)	54.50%

RCTs=randomized controlled trials; mCPR=mechanical CPR; N/A=not available
